# Application of the geographic population structure (GPS) algorithm for biogeographical analyses of wild and captive gorillas

**DOI:** 10.1186/s12859-018-2568-5

**Published:** 2019-02-05

**Authors:** Ranajit Das, Priyanka Upadhyai

**Affiliations:** 1 Manipal Centre for Natural Sciences (MCNS), Manipal Academy of Higher Education (MAHE), University building, Lab 11, Madhav Nagar, Manipal, Karnataka 576104 India; 2 Department of Medical Genetics, Kasturba Medical College, Manipal Academy of Higher Education, Manipal, Karnataka India

**Keywords:** Biogeography, Admixture, Geo-localization of non-human species, Gorilla ancestry

## Abstract

**Background:**

The utilization of high resolution genome data has important implications for the phylogeographical evaluation of non-human species. Biogeographical analyses can yield detailed understanding of their population biology and facilitate the geo-localization of individuals to promote their efficacious management, particularly when bred in captivity. The Geographic Population Structure (GPS) algorithm is an admixture based tool for inference of biogeographical affinities and has been employed for the geo-localization of various human populations worldwide. Here, we applied the GPS tool for biogeographical analyses and localization of the ancestral origins of wild and captive gorilla genomes, of unknown geographic source, available in the Great Ape Genome Project (GAGP), employing Gorillas with known ancestral origin as the reference data.

**Results:**

Our findings suggest that GPS was successful in recapitulating the population history and estimating the geographic origins of all gorilla genomes queried and localized the wild gorillas with unknown geographical origin < 150 km of National Parks/Wildlife Reserves within the political boundaries of countries, considered as prominent modern-day abode for gorillas in the wild. Further, the GPS localization of most captive-born gorillas was congruent with their previously presumed ancestral homes.

**Conclusions:**

Currently there is limited knowledge of the ancestral origins of most North American captive gorillas, and our study highlights the usefulness of GPS for inferring ancestry of captive gorillas. Determination of the native geographical source of captive gorillas can provide valuable information to guide breeding programs and ensure their appropriate management at the population level. Finally, our findings shine light on the broader applicability of GPS for protecting the genetic integrity of other endangered non-human species, where controlled breeding is a vital component of their conservation.

**Electronic supplementary material:**

The online version of this article (10.1186/s12859-018-2568-5) contains supplementary material, which is available to authorized users.

## Background

The importation of western gorillas (*Gorilla gorilla*) from their native habitat in Africa to North American zoos began over 100 years ago [1]. While most wild gorillas transferred initially died shortly after arrival [2, 3], those introduced subsequently between the 1930s and 1970s survived for several decades [3]. Overall, at least 283 wild gorillas have been imported to North America [4]. However, since their inclusion under the protection of the Convention on International Trade in Endangered Species of Wild Fauna and Flora (CITES) in 1975 there have been no wild born gorillas added to the captive population. Notably for a majority of the gorillas in captivity sufficient information pertaining to their biogeographic origin is unavailable [5]. Gorillas were pronounced as critically endangered in 2007 [6]; in the wild their population is rapidly dwindling owing to severe habitat encroachment, the illegal bushmeat trade and susceptibility to diseases such as Ebola. The limited availability of information regarding the biogeographic ancestry of gorillas has likely constrained their management pertaining to maximizing genetic diversity at the species level, which can be achieved by preventing inbreeding among related individuals. It is noteworthy that unlike in the wild, captive gorillas have been revealed as significantly more admixed from two or more genetically distinct wild born populations [1, 4, 7].

Given the strong correspondence between geography and genetics [8, 9], a number of strategies have focused on the delineation of the precise geographic origin of human populations using high-resolution genetic data. The Geographic Population Structure (GPS) algorithm is an admixture based tool that has so far been employed for the biogeographical analyses of human populations and is likely superior to other existing methods for the same [9–13]. It has been successfully used to reconstruct history of several human populations worldwide [9, 13–19]. In brief, it deduces the genomic proximity between the query and reference individuals to determine the likely biogeographical affinity of the former using the geographic coordinates (latitude and longitude) corresponding to the latter as reference.

Here we aimed to assess whether the GPS algorithm, essentially designed for biogeographical analyses of human populations could be applied to non-human species with equal precision and efficiency. We investigated the whole genome sequence (WGS) information from 31 gorilla genomes available in Great Ape Genome Project (GAGP) [7] corresponding to two subspecies of western gorillas (*Gorilla gorilla*), namely western lowland gorilla (*Gorilla gorilla gorilla*) and Cross River gorilla (*Gorilla gorilla dielhi*), as well as the eastern lowland gorilla (*Gorilla beringei graueri*); using the GPS tool we localized the ancestral origins of both wild and captive gorillas of unknown geographic origins, employing those with a known provenance, as reference. Our findings suggest that GPS was successful in inferring the geographic origins and recapitulating the population history of the gorilla genomes queried. It uncovers the broader utility of biogeographical analyses tools, in particular GPS, to facilitate deeper insight into the population biology of endangered non-human species that can foster their efficacious management and conservation.

## Results

### Clustering of populations and admixture analysis

**Fig. 1:**
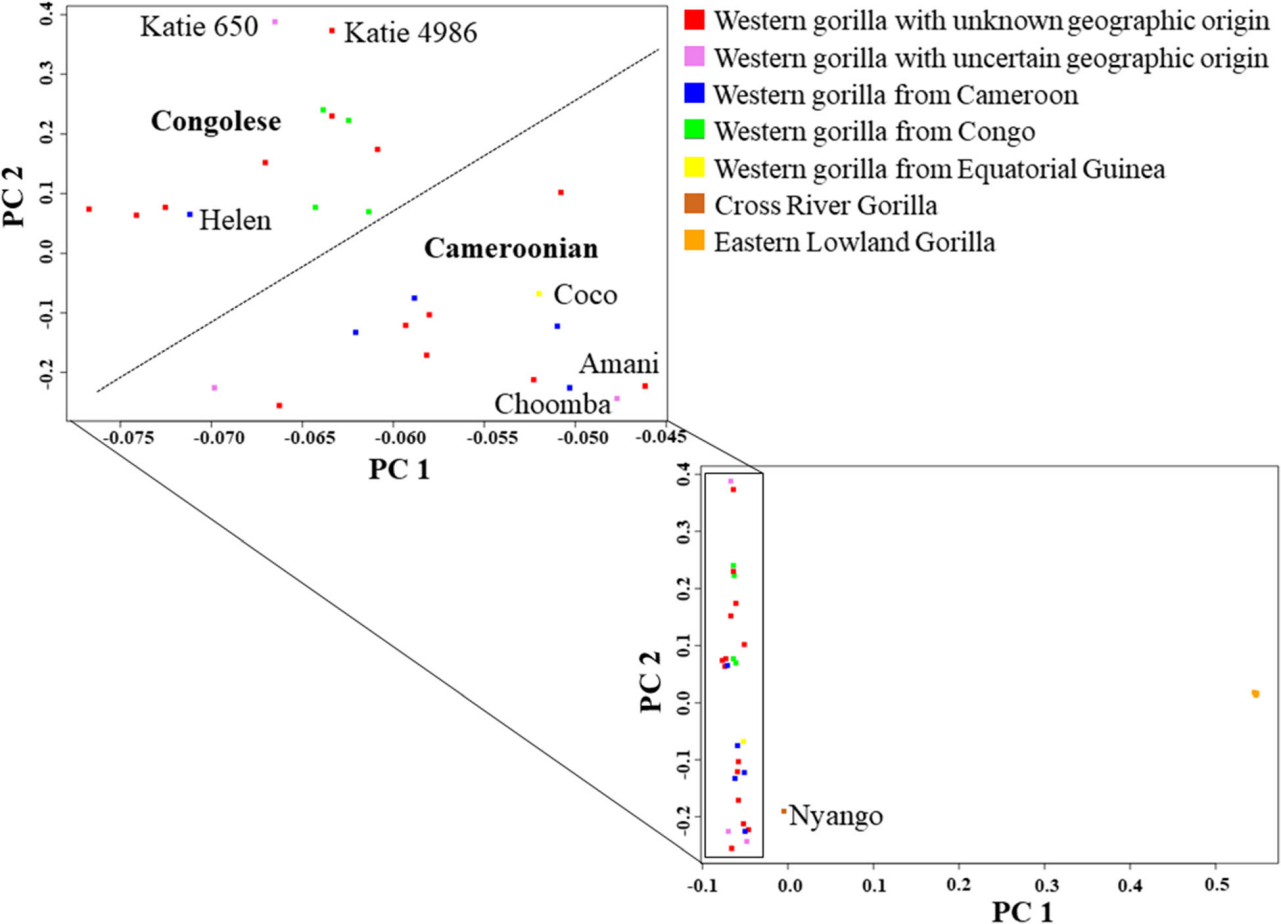
Principal Component Analysis (PCA) of gorilla genomes. PCA plot showing genetic differentiation among gorilla genomes. PCA was performed in PLINK v1.9 and the top four principal components (PCs) were extracted. Top two PCs (PC1 and PC2), explaining the highest variance of the data were plotted in R v3.2.3. The colors of the gorilla genomes in the PCA plots corresponds to the location geographic location, they belonged to. The red squares represent gorilla genomes with unknown geographic origin. The X-axis (PC1), depicting eastern-western gorilla differentiation, explains 45% variance while the Y-axis (PC2), indicating clustering among western gorillas, explains 23% variance of the data

Principal Component Analysis (PCA) was performed in PLINK v1.9 and the top two PCs were plotted in R v3.2.3. Our PCA results concur with previous observations of an eastern gorilla - western gorilla contrast along the horizontal principal component (PC1) and vertical differentiation (PC2) among western gorilla genomes [7] (Fig. 1). Two distinct clusters were found among western gorillas along PC1: one predominantly composed of Cameroonian gorillas and the other largely consisted of Congolese gorillas. Notably, Coco whose birthplace was recorded as Equatorial Guinea [7] appeared to group with Cameroonian gorillas, owing to its high genomic proximity with them. This likely alludes to the substantial genomic affinity of gorillas from Equatorial Guinea and southwest Cameroon. However, validation of the same would be feasible only with the availability of high resolution genomic data of other gorillas, from Equatorial Guinea. Among gorillas with unknown birthplace information, Katie (B650) and Katie (KB4986) were clustered at one extreme of Congolese-Cameroonian cline, while Choomba and Amani appeared at the other end.

**Fig. 2:**
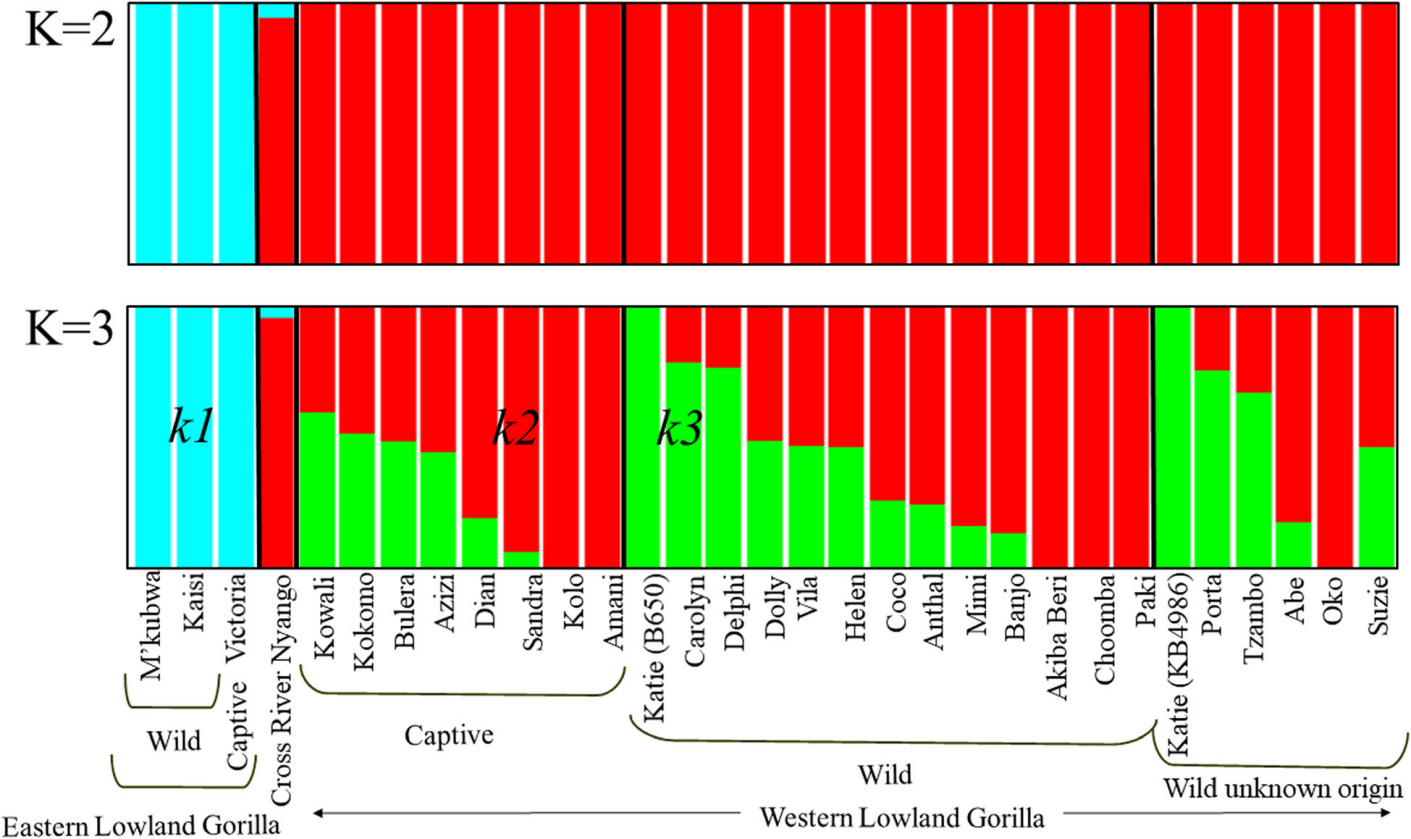
*Admixture analysis of gorilla genomes.* Admixture plots showing the ancestry components of gorilla genomes. Admixture proportions were generated through an unsupervised admixture analysis at *k* = 2 and *k* = 3 using ADMIXTURE v1.3 and plotted in R v3.2.3. Each individual is represented by a vertical line partitioned into colored segments whose lengths are proportional to the contributions of the ancestral components to the genome of the individual. At *k* = 2, with lowest cross-validation error, eastern and western gorillas were homogeneously assigned to two distinct clusters. Nyango, the cross-river gorilla, expectedly had high western gorilla ancestral component with small fraction of eastern gorilla ancestry. At *k* = 3, while the eastern gorillas maintained their genomic integrity, most western gorillas appeared to be genomic admixture of Cameroonian and Congolese ancestral components of varying proportions

At *K = 3*, the eastern lowland gorillas were homogeneously assigned to a unique cluster (*k1*) while most western gorillas appeared to be a genomic admixture of Cameroonian (*k2*) and Congolese (*k3*) components in varying proportions (Fig. 2). Interestingly, the wild-born Akiba-Beri, Choomba, Paki, Oko, and the captive-born Kolo and Amani have maintained their genomic integrity, such that their entire genome consisted of the Cameroonian admixture component. Similarly, Katie (B650) and Katie (KB4986) also appeared as pure-bred and are composed of the Congolese admixture component.

### Biogeographical mapping of reference gorillas

**Fig. 3:**
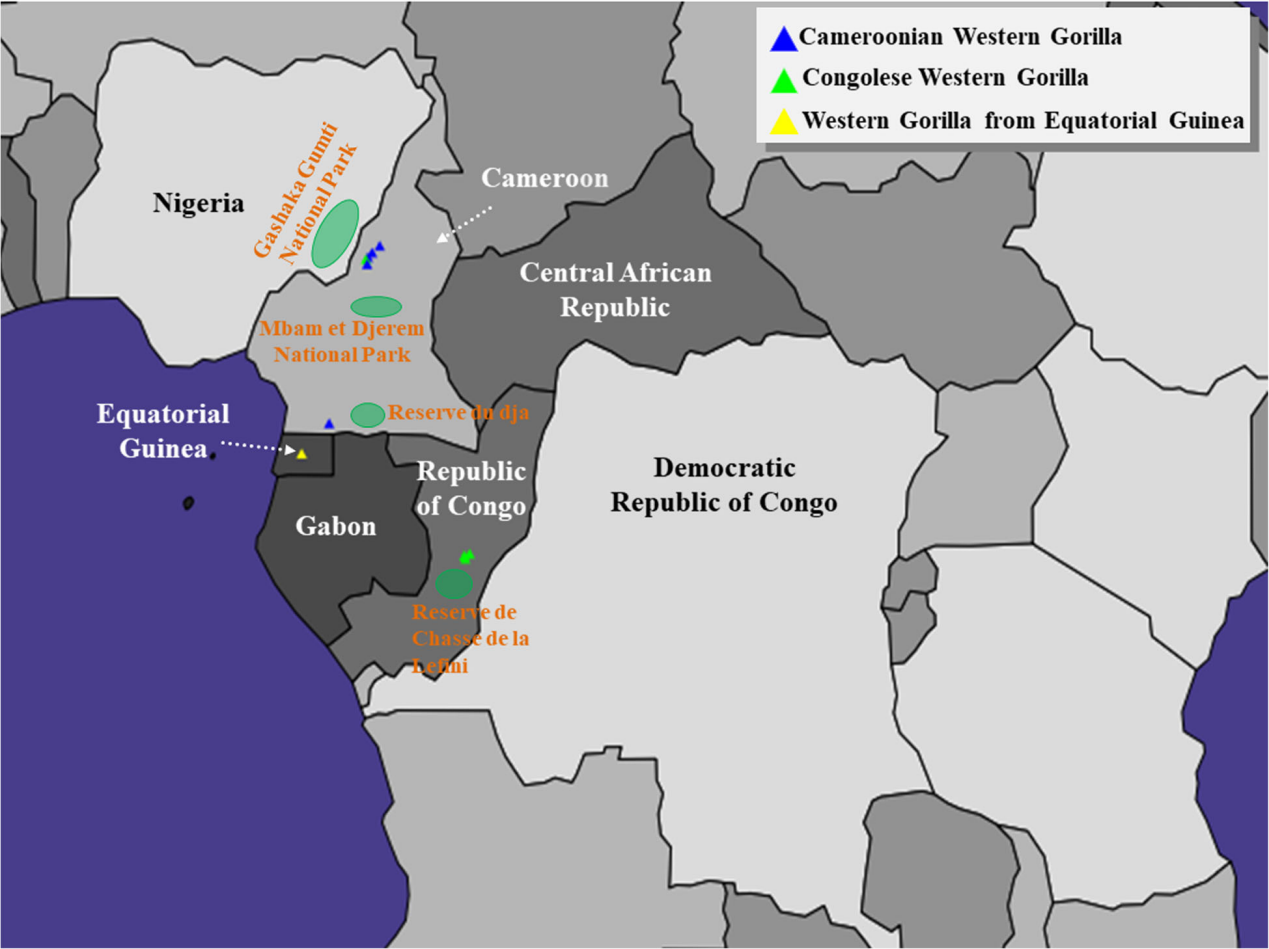
GPS prediction of the biogeographical affinities of gorilla populations of known geographic source from the Great Ape Genome Project (GAGP). Relevant National Parks/Wildlife Reserves are shown in green ovals (not to scale). The geographic coordinates ascertained by GPS for gorillas from Equatorial Guinea, Cameroon and Congo are shown in yellow, blue and green triangles, respectively. Maps were plotted using the R package rworldmap v1.3–1

Prior to applying GPS to elucidate the biogeographical affinity of the query gorilla genomes with unknown geographical origin, we sought to trial its accuracy for all ten reference western lowland gorillas, of known geographic origins [7]. Assignment accuracy was determined for each individual based on whether the predicted geographical coordinates localized within the political boundaries of the known country or regional location of origin. We note that GPS assignments were consistent with the recorded geographic source for nine out of ten individuals assessed. It positioned Coco to Equatorial Guinea, all Cameroonian gorillas to Cameroon and three out of four Congolese gorilla to Congo (Fig. 3). One Congolese gorilla, Vila, did not correspond to its documented ancestral origin, instead it was positioned with other Cameroonian gorillas near Pangar Djerem Researve, Mbam et Djerem National Park, Cameroon, which is home to the northernmost known population of the western lowland gorillas. This mismatch in the assignment of Vila could be likely attributed to its high genomic proximity with a Cameroonian gorilla, Helen (Fig. 3). Overall these results demonstrate a strong geographic-genomic correlation and delineate the expected assignment error for our analyses.

We note that due to the unavailability of geographic coordinates of the reference gorillas within their ancestral countries, the precise prediction accuracy (in km) of the gorillas with known origin was not possible.

### Biogeographical mapping of gorillas with unknown geographic origin

**Fig. 4:**
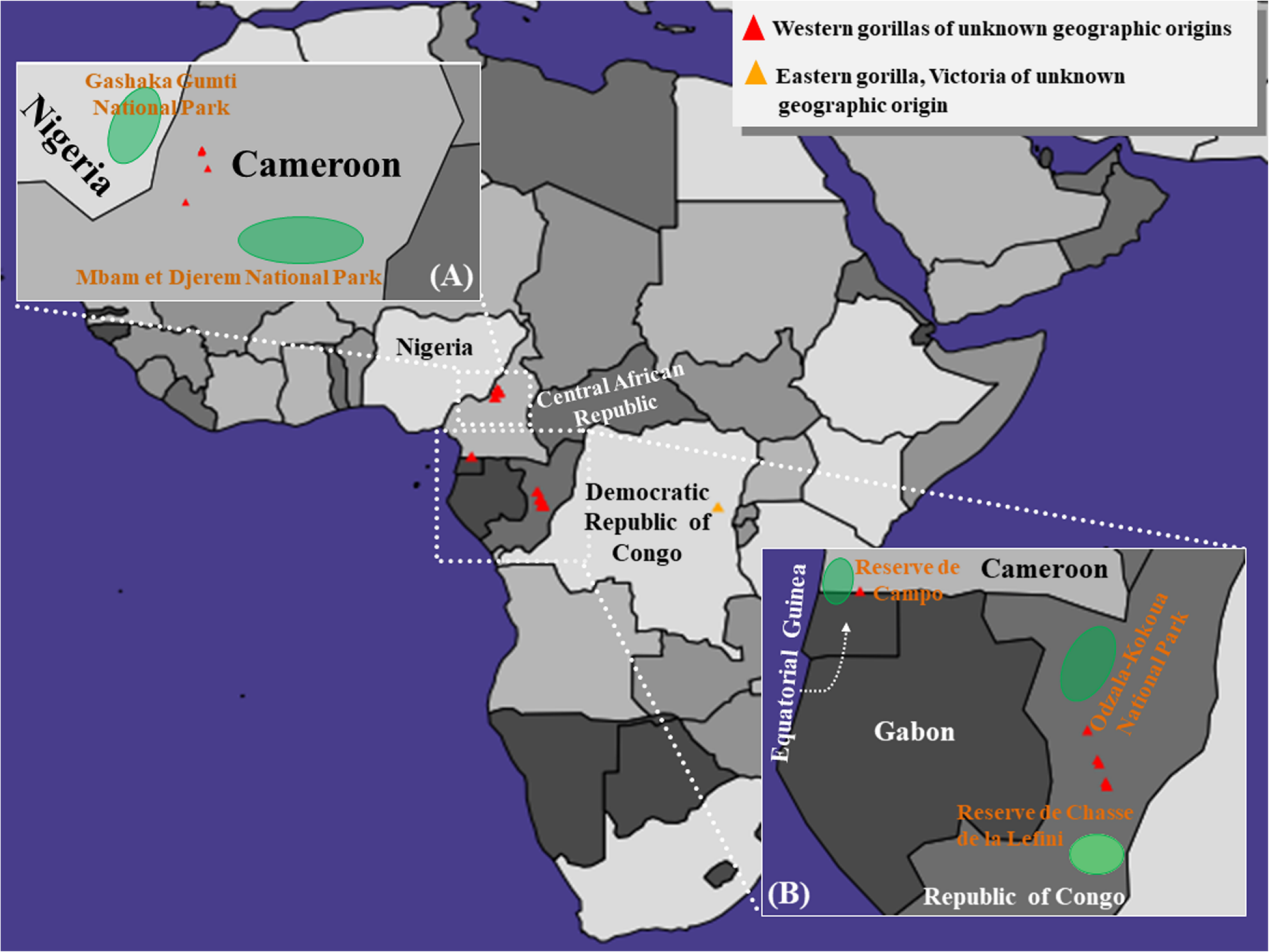
GPS predictions for the gorillas of unknown geographic origin from the Great Ape Genome Project (GAGP). A map depicting the GPS predicted locations for gorillas with unknown geographic origin. The red and orange triangles depict western gorillas and eastern lowland gorillas, respectively of unknown geographic origins. Relevant National Parks/Wildlife Reserves are shown in green ovals (not to scale). *Note*: in some cases, multiple individuals were assigned to the same geographic location and therefore appeared as a single individual. Maps were plotted using the R package rworldmap v1.3–1

Next, we applied the GPS algorithm to infer the biogeographical affinity of 18 query gorillas of unknown provenance (Fig. 4). It is noteworthy that eastern gorilla populations are known to occur in the Democratic Republic of Congo, Uganda, and Rwanda, whereas western gorilla populations reside primarily in Cameroon, Equatorial Guinea, Gabon, Congo, and the Central African Republic [20]. In agreement with their known homelands, GPS positioned all western lowland gorilla genomes within Equatorial Guinea, Cameroon and in the Republic of Congo, while, Victoria, an eastern lowland gorilla, born in captivity was assigned to east-central Democratic Republic of Congo (Additional file 1 Table S3).

Specifically, among the wild-born western lowland gorillas of unknown birthplace data, Abe, Oko, Choomba, Paki, and Suzie were assigned to central Cameroon, 100–150 km from Mbam et Djerem National Park. While Tzambo was positioned in Central Congo, halfway between Odzala-Kokoua National Park and Reserve de Chasse de la Lefini; Porta, Katie (B650) and Katie (KB4986) were positioned < 100 km from Reserve de Chasse de la Lefini.

Among the captive-born western lowland gorillas, Kowali, Kokomo and Bulera were placed in Congo. The remaining individuals, Azizi, Kolo, Amani, and Sandra all were positioned in Cameroon. Finally, Dian was positioned in southwest Cameroon on the border of Cameroon and Equatorial Guinea. Interestingly, the GPS localization of captive-born gorillas, Victoria, Dian, Sandra, Kolo, and Azizi was congruent with their previously presumed ancestral homes [7].

Further, to test whether presence of close relatives have any impact on the outcome of GPS, we repeated the analysis discarding Bulera, Kowali, Suzie and Oko, which were identified as 1st, 2nd or 3rd order relatives of the reference gorillas based on their genomic information [7]. We found that there is no discernible effect of ‘relationship’ in the outcome of GPS analysis. GPS positioned the remaining individuals at the same geographical coordinates as it did before (without exclusion of the relatives).

### reAdmix analysis

Since gorillas born in captivity have been revealed as discernibly more admixed when compared to wild born populations [1, 4, 7] and GPS is limited in its ability to localize highly admixed individuals [15, 21], we interrogated nine captive gorillas (one eastern lowland gorilla and eight western lowland gorillas) via reAdmix analysis [21], to estimate the composition of captive gorillas as a weighted mix of wild populations (Table 1). Azizi and Bulera were found to be highly admixed with higher Cameroonian (48.5 and 54.4% respectively) compared to Congolese (46.1 and 36.1% respectively) admixture proportions. While the GPS tool appropriately localized Azizi to Cameroon, it failed to do so for Bulera, reflecting that the predictive precision for GPS is curtailed in case of highly admixed individuals. Among others, one or more wild ancestral populations are likely missing in this study, for Kowali and Kokomo. However, both are assigned to Congo, potentially based on their genomic proximity to Congolese gorillas. Finally, Sandra, Kolo, Amani, and Dian had significantly high Cameroonian admixture proportions (86–100%) and were the least admixed, among the captive gorillas included in this study, likely leading them to be positioned with enhanced precision.

**Table 1:** Admixture proportions of various captive gorillas employed in this study, as revealed by reAdmix analysis

Name	Potential origin	Fractions	Comment
Victoria	Congo_1	1	
Kowali	West Africa_1	0.411	Some wild populations from where it likely originated are missing in the current study
Azizi	West Africa_1	0.177	
	West Africa_2	0.147	
	Equatorial_Gunea_1	0.034	
	Cameroon_2	0.308	
	Cameroon_1	0.02	
	Congo_3	0.314	
Bulera	West Africa_1	0.192	
	West Africa_2	0.09	
	Cameroon_2	0.352	
	Congo_3	0.357	
	Congo_2	0.009	
Kokomo	Congo_1	0.013	Some wild populations from where it likely originated are missing in the current study
	West Africa_2	0.475	
Sandra	West Africa_2	0.059	
	West Africa_1	0.939	
Dian	West Africa_2	0.119	
	Cameroon_1	0.855	
Kolo	West Africa_1	1	
Amani	West Africa_1	0.998	
	Congo_1	0.002	

The reference populations mentioned in the ‘Potential origin’ column has been generated through *leave-one-out* procedure using the genomic information of gorilla individuals with known geographic origins. The ‘Fractions’ depict the ancestry proportion of the query individuals for each of the reference populations

## Discussion

Great apes such as the gorillas are faced by serious challenges impacting their population size and distribution, in the wild. This has translated into increasing focus on their preservation both in the wild as well as in captivity. While management of gorilla breeding programs in North America have afforded prominent impetus to maximizing genetic diversity in order to avoid inbreeding depression, they have been limited by sufficient information of the phylogeographic ancestries of the individuals bred in captivity. As a result, the analyses of captive born gorillas in North American zoos and sanctuaries has revealed them with high genetic heterozygosity due to admixture between two or more genetically distinct wild born populations leading to an attenuation of the phylogeographic signal [1, 4, 7]. Presently there is limited knowledge of the ancestral origins of founders of most North American captive gorillas [5]. Hence, determination of the native geographical source of captive gorillas can be a valuable tool to foster their population level management.

Here we sought to evaluate whether the GPS algorithm, largely employed for biogeographical analyses of human populations [9, 13–16] could be applied to non-human species, and to estimate its efficacy in doing the same. We applied the GPS tool to interrogate available gorilla genomes [7] and estimated the ancestral biogeographic affinities of 18 query captive and wild born gorillas, of unknown source.

Inference of the biogeographic proximity of individuals, based on genetic data has been challenging and of interest to biologists over decades. The GPS tool correlates the relative proportions of admixture in the query and reference individuals to deduce the likely geographic location of the former based on the geographic coordinates of the latter. Here, we note that our present findings are based on the coordinates corresponding to the geographic centers of the countries, where the reference individuals are documented to have originated, owing to the unavailability of precise regional locations for the same. Despite this, our trial analyses successfully assigned nine out of ten gorillas queried within the countries recorded as their places of birth, reflecting an acceptable genomic-geographic correspondence and reliable predictive accuracy. Further affirmation to the utility of our methods is evidenced in the assignment of the captive-born gorillas, Victoria, Dian, Sandra, Kolo, and Azizi, to locations within countries consistent with their previously inferred ancestral homes [7]. For query western lowland gorilla genomes of unknown source, GPS localized their ancestral origins < 150 km of the National Parks/Wildlife Reserves within the political boundaries of the countries, Equatorial Guinea, Cameroon and the Republic of Congo that are considered as prominent modern-day habitat for western lowland gorillas in general [20] (Fig. 4).

These findings also largely resonated with previous mitochondrial haplogroup analysis (Additional file 1 Figure S1) [22]; GPS positioned Abe, Paki, and Oko of haplogroup C1 to central Cameroon and localized gorillas of haplogroup D3, Bulera, Kowali, Porta, and Katie to Congo, consistent with their previously deduced phylogeographic origins [22]. Interestingly, Azizi with mtDNA haplogroup D3, which is predominantly found in Congo [22], was assigned to Cameroon and this was further supported by the results of reAdmix analysis (Table 1) that estimated a prominent Cameroonian admixture proportion for Azizi. This potentially reflects the pedigree of Azizi, whose mother was from Congo and both father and maternal grandfather was from Cameroon. Thus, it can be surmised that while her mtDNA derives from Congo, the majority of her nuclear genome would reflect Cameroonian ancestry. Finally, our current assignment of Kokamo (haplogroup D2) to Congo and Choomba (haplogroup C3) to central Cameroon also coincided with previous results, mentioning that C3 is distributed in central Cameroon along the south bank of the Sanaga River and D2 is found in the Dzanga-Sangha region of Central African Republic, along the border of Congo. [22].

It is noteworthy that our results positioned some western lowland gorillas, in close proximity (< 50 km) to Gashaka Gumti National Park located in central Cameroon, on its border with Nigeria, in a region that is home to Nigerian chimpanzees and where gorillas have likely never been found. We surmise that this reflects an underlying limitation of the GPS strategy, which is strongly guided by the availability of appropriate reference data, such that our assumption of the geographic centers of countries corresponding to the reference dataset, likely drew the query individuals in this case, farther north from their known southern Cameroonian homeland. Similarly, it is noteworthy that our findings are bereft of any query gorillas being assigned to Gabon, a known major natural homeland for western lowland gorillas in the wild, likely due to the absence of suitable genomic references for the same.

Given that the GPS framework is more error-prone for highly admixed individuals [9, 13, 21] we sought to improve our resolution into the biogeographical ancestry of the captive gorillas in our query pool, known to be admixed, by evaluating them using reAdmix [21]. Out of the two most admixed individuals, Azizi and Bulera (Table 1), GPS successfully localized the former to its previously presumed ancestral home, Cameroon, but failed to do so for the latter, manifesting its inherent limitation in interpreting highly mixed individuals. Nonetheless, it assigned the least admixed captive gorillas, Sandra, Kolo, Amani, and Dian, to locations concurrent with their earlier inferred ancestral homes (Fig. 4) [7].

Our findings from PCA and ADMIXTURE suggested two prominent population clusters amidst the western lowland gorillas, this is not only reminiscent of previous studies, based on high resolution WGS [7] and microsatellite data [4, 23], but provided enhanced insight into the same. A case in point would be the grouping of Coco, whose birthplace was documented as Equatorial Guinea [7] with Cameroonian gorillas, due to its high genomic affinity with the latter. We surmise this could likely reflect the substantial genomic similarity of gorillas from Equatorial Guinea and southwest Cameroon and that they may constitute one panmictic population. However, a more conclusive understanding of the same would only be achieved with the availability of genomic data of other gorillas from Equatorial Guinea. We also note that a previous WGS based study did not identify any substructure among western lowland gorillas. This is likely because of limited knowledge regarding biogeographical affiliation of gorillas with unknown ancestral home (both captive born and wild born but unknown origin) [24]. However, similar to the present study, it could delineate two distinct clusters of western gorillas along PC1 and could deduce close affinities between gorillas from Cameroon and Equatorial Guinea and one Cameroonian gorilla (likely Helen) with Congolese gorillas.

## Conclusions

The utilization of high resolution genomic information has important implications for the phylogeographical evaluation of non-human species such as the great apes. Effective conservation of captive and wild populations of gorillas necessitates the delineation of the biogeographic affinities of their founders, so as to facilitate preservation of the population level integrity of genomic signal. This could be particularly relevant for planned introduction of animals, such as those being carried out in Central Africa [25]. Given this context, the current findings revealed the GPS algorithm to function with reasonable accuracy in localizing the ancestral source of gorilla genomes queried, to the countries which constitute their natural homeland, in the wild. When interpreted with adequate caution against the inherent limitations of the GPS tool, these results recapitulate and expand upon previous studies [4, 7, 22] to yield a better insight into the genetic relatedness and biogeography of the gorilla genomes assessed here. To the best of our knowledge this is the first application of the GPS algorithm for interrogating gorilla genomes, of unknown provenance and underscores its broader applicability for geo-localization of other endangered non-human species, particularly those bred in a controlled manner, to bolster their efficient management and conserve their genetic integrity.

## Method

### Dataset

The dataset employed in this study comprised of 31 gorilla genomes available in Great Ape Genome Project (GAGP) [7]: western lowland gorilla (*Gorilla gorilla gorilla*, *N* = 27), eastern lowland gorilla (*Gorilla beringei graueri, N* = 3), and Cross River gorilla (*Gorilla gorilla dielhi*, *N* = 1) (Additional file 1 Table S1). The geographic origins of all individuals denoted here are as per those indicated previously [7]. Most captive gorillas have been demonstrated as highly genetically heterogeneous, when compared to wild born individuals, as a consequence of being admixed from two or more genetically distinct wild born populations [1, 7]. Therefore, we considered them to be of unknown geographic origins, regardless of their recorded geographic source. Overall, 13 wild born gorillas with known birthplace information [7] were deemed to be of known geographic origins, while the remainder whose provenance was ambiguous or undefined were considered to be of unknown origin. We note that while the documented provenance data for wild born gorillas may be largely accurate, nevertheless, discrepancies in cataloguing this information could have occurred in a minority of the cases, and therefore adequate caution is warranted in interpreting the consequent findings. The variant calling file (VCF) was obtained from the GAGP database (http://biologiaevolutiva.org/greatape/data.html). It was converted into PLINK .ped format using VCFtools v.0.1.13 [26]. We used quality control filters as employed previously for this dataset [7]. A pruned subset of Single Nucleotide Polymorphisms (SNPs) that are in linkage equilibrium with each other was generated using --indep-pairwise function in PLINK v1.9 (https://www.cog-genomics.org/plink/1.9/) [27]. During pruning, the window size for SNPs was kept at 50, 5 SNPs were allowed to shift the window at each step, and the r^2^ threshold was kept at 0.1 (−-indep-pairwise 50 5 0.1). Further, we used 0.05 as MAF threshold and 0.1 as missing genotype threshold by employing --maf 0.05 and --geno 0.1 flags respectively as additional quality control measures. Before pruning the raw dataset comprised of 53,178,815 SNPs. Among them, 52,824,735 SNPs were pruned out for being in linkage disequilibrium (r^2^ > 0.1) generating to a final dataset of 354,080 SNPs.

### Population clustering and admixture analysis

Principal component analysis (PCA) was performed in PLINK v1.9 using --pca command. The top four principal components (PCs) of the variance-standardized relationship matrix were extracted and the top two of the same were plotted.

The ancestry of the gorilla genomes was estimated using unsupervised clustering as implemented in ADMIXTURE v1.3 [28]. Admixture analyses were performed for *K = 2* and *K = 3* as done previously [7]. Despite Cross validation metric (CVE) [28] indicating that *K = 2* has the lowest error rate, we chose *K = 3* for further analysis so as to accentuate our ability to resolve the western gorilla genomes into the Congolese and Cameroonian clusters. PCA and Admixture plots were generated in R v3.2.3.

### Biogeographical mapping of gorilla genomes

Biogeographical analysis was performed using the Geographic Population Structure (GPS) algorithm as described previously [9]. The GPS algorithm correlates the admixture patterns of individuals of unknown origins using the admixture fractions (GEN file) and geographical locations or coordinates (GEO file) of reference individuals with known geographical origin. Given samples of unknown geographic origin and admixture proportions that correspond to putative ancestral populations, GPS can convert the genetic distances between the query and the most proximal reference populations into geographic distances. Comparing the admixture proportions of the query with the reference populations, it extrapolates the genomic similarity of the former and infers its geographic origins using the known biogeographical information of the reference. Here we curated the reference gorilla dataset using the ‘*leave-one-out*’ procedure at the individual level, as described previously [9] using all available gorillas with a known geographical source.

First we sought to trial the accuracy of GPS mediated biogeographical analyses using gorilla genomes of known provenance. Accordingly, we analyzed the genomic information pertaining to ten western lowland gorillas from Cameroon, Republic of Congo, and Equatorial Guinea (Additional file 1 Table S1) and estimated their admixture proportions with respect to the three admixture components corresponding to the reference gorilla genomes.

Subsequently we mapped 18 gorilla genomes (17 western lowland gorilla and one eastern lowland gorilla) of unknown geographical origin with respect to the reference dataset, and interpreted their admixture fractions and geographic locations (latitudinal and longitudinal coordinates). Therefore, our GEN file contained three admixture coefficients corresponding to the reference genomes and the GEO file contains the associated geographic coordinates (latitude and longitude). We note that given the unavailability of precise geographical locations for our reference dataset, the coordinates for the geographic centers of the countries where the reference individuals originated, namely Cameroon, Republic of Congo, and Equatorial Guinea [7], were employed for our analyses (Additional file 1 Table S2).

### reAdmix analysis

Given that the GPS tool is likely less efficient in interpreting the biogeographical affinity of highly admixed individuals [15, 21] and since our query dataset consists of captive gorillas, known to be discernibly genetically more admixed from two or more wild populations [1, 4, 7], we supplemented our analyses by using the reAdmix algorithm [21]. reAdmix treats the tested individual and *N* reference populations as points inside the standard simplex in K-dimensional space of admixture proportions [21]. It represents the tested individual T as a convex combination reference ancestries: $T=\sum \limits_{i=1}^N{w}_i{R}_i$, where *R*_*i*_ are admixture vector of the i^th^ reference population, and *w*_*i*_ is its contribution. reAdmix tool has been effectively used in estimating biogeographical origin of highly admixed individuals [18, 19, 21].

We used the same admixture vectors in the K = 3 dimensional space as we used for the GPS analysis. We have four super-populations of wild western lowland gorillas [7]: Congo, Cameroon, Equatorial Guinea, and West Africa. However, the wild gorillas are not genetically homogeneous within these large regions, and we have identified genetically validated groups of reference individuals within each super-population. There were three groups in Congo, two in Cameroon, and two in West Africa. The groups are denoted by a subscript (e.g. Cameroon_2). These reference populations were used to estimate proportions of wild ancestries in all nine captive gorillas of unknown origin. Note that the cases where the admixture proportions do not sum to 100% could be likely attributed to the absence of one or more wild ancestral populations in the reference dataset. reAdmix analysis (see Table 1) confirms that Victoria is a pure Congo gorilla, Kolo and Amani are predominantly West African, while Azizi and Bulera are highly admixed.

## Additional file

Additional file 1 : **Table S1.** Biogeographic information about the gorilla individuals employed in current study. **Table S2.** Latitudes and Longitudes of reference gorillas. **Table S3.** Latitudes and Longitudes of query gorillas. **Figure S1.** Distribution of mtDNA haplogroups among the gorilla genomes used in the current study (after Soto-Calderon et al. [23]). (DOCX 41 kb)
