# Supplementary material for: Application of the geographic population structure (GPS) algorithm for biogeographical analyses of wild and captive gorillas
Source: BMC Bioinformatics. 2019 Feb 5;20(Suppl 1):35. doi: 10.1186/s12859-018-2568-5 (PMC6362561; doi:10.1186/s12859-018-2568-5)
Supplement: Supplementary file 1 — : Table S1. Biogeographic information about the gorilla individuals employed in current study. Table S2. Latitudes and Longitudes of reference gorillas. Table S3. Latitudes and Longitudes of query gorillas. Figure S1. Distribution of mtDNA haplogroups among the gorilla genomes used in the current study (after Soto-Calderon et al. [23]). (DOCX 41 kb) [file 12859_2018_2568_MOESM1_ESM.docx]

**Supplemental Table 1**: Biogeographic information about the gorilla individuals employed in current study

| **Species** | **Common name** | **Name** | **Studbook ID** | **Sex** | **Geographic origin^1^** | **Birth Origin^[1]^** | **Categorization used in this study** | **Mitochondrial Haplogroup affiliation^[2]^** |
| --- | --- | --- | --- | --- | --- | --- | --- | --- |
|  |  | Carolyn | 3 | F | Congo | Wild born | Known | - |
|  |  | Abe | 52 | M | Unknown | Wild born | Unknown | C1 |
|  |  | Porta | 64 | F | Unknown | Wild born | Unknown | D3 |
|  |  | Vila | 80 | F | Congo | Wild born | Known | D3 |
|  |  | Helen | 96 | F | Cameroon | Wild born | Known | D3 |
|  |  | Choomba | 180 | F | West Africa | Wild born | Unknown | C3 |
|  |  | Paki | 191 | F | West Africa | Wild born | Unknown | C1 |
|  |  | Oko | 192 | F | Unknown | Wild born | Unknown | C1 |
|  |  | Dolly | 195 | F | Congo | Wild born | Known | D2 |
|  |  | Delphi | 230 | F | Congo | Wild born | Known | - |
|  |  | Mimi | 241 | F | Cameroon | Wild born | Known | - |
|  |  | Banjo | 255 | M | Cameroon | Wild born | Known | - |
| *Gorilla gorilla gorilla* | Western lowland gorilla | Tzambo | 440 | M | Unknown | Wild born | Unknown | - |
|  |  | Katie (KB4986) | 498 | F | Unknown | Wild born | Unknown | D3 |
|  |  | Katie (B650) | 498 | F | West Africa | Wild born | Unknown | D3 |
|  |  | Suzie | 636 | F | Unknown | Wild born | Unknown | - |
|  |  | Kowali | 663 | F | Unknown | Captive born | Unknown | D3 |
|  |  | Amani | 899 | F | Unknown | Captive born | Unknown | - |
|  |  | Kolo | 936 | F | Cameroon | Captive born | Unknown | - |
|  |  | Sandra | 969 | F | Cameroon | Captive born | Unknown | - |
|  |  | Kokamo | 1049 | F | Unknown | Captive born | Unknown | D2 |
|  |  | Dian | 1091 | F | Cameroon | Captive born | Unknown | - |
|  |  | Bulera | 1120 | F | Cameroon | Captive born | Unknown | D3 |
|  |  | Coco | 1351 | F | Equatorial Guinea | Wild born | Known | - |
|  |  | Azizi | 1459 | M | Cameroon | Captive born | Unknown | D3 |
|  |  | Akiba Beri | 1926 | F | Cameroon | Wild born | Known | - |
|  |  | Anthal | 1930 | F | Cameroon | Wild born | Known | - |
|  |  | M'kubwa | 9907 | M | DRC - Tulakwa, Northwest of Bukavu | Wild born | Known | - |
| Gorilla beringei graueri | Eastern lowland gorilla | Kaisi | 9909 | M | DRC - Walikale region, Nord-Kivu | Wild born | Known | - |
|  |  | Victoria | 9919 | F | DRC | Captive born | Unknown | - |
| Gorilla gorilla diehli | Cross River gorilla | Nyango | 9941 | F | Cameroon | Wild born | Known | - |

1. Prado-Martinez J, Sudmant PH, Kidd JM, Li H, Kelley JL, Lorente-Galdos B, Veeramah KR, Woerner AE, O'Connor TD, Santpere G *et al*: **Great ape genetic diversity and population history**. *Nature* 2013, **499**(7459):471-475.

2. Soto-Calderon ID, Dew JL, Bergl RA, Jensen-Seaman MI, Anthony NM: **Admixture between historically isolated mitochondrial lineages in captive Western gorillas: recommendations for future management**. *J Hered* 2015, **106**(3):310-314.

**Supplemental Table 2**: Latitudes and Longitudes of reference gorillas

| **Country** | **Latitude** | **Longitude** | **Comments** |
| --- | --- | --- | --- |
| Democratic Republic of Congo | -1.57 | 27.95 | Coordinates of Tulakwa, birthplace of M'kubwa |
| Republic of Congo | -1.44 | 15.56 | Geographic centre of the country, as provided by Google Earth |
| Cameroon | 7.37 | 12.35 | Geographic centre of the country, as provided by Google Earth |
| Equatorial Guinea | 1.65 | 10.27 | Geographic centre of the country, as provided by Google Earth |

**Supplemental Table 3**: Latitudes and Longitudes of query gorillas

| **Individual** | **GPS predicted Latitude** | **GPS predicted Longitude** |
| --- | --- | --- |
| Victoria | -1.5700 | 27.9500 |
| Kowali | -0.3812 | 15.1632 |
| Azizi | 7.1808 | 12.4188 |
| Bulera | -1.3751 | 15.5364 |
| Kokomo | -1.0197 | 15.4063 |
| Dian | 2.2766 | 10.5016 |
| Kolo | 7.3700 | 12.3500 |
| Amani | 7.3700 | 12.3500 |
| Sandra | 7.3491 | 12.3438 |
| Suzie | 7.3692 | 12.3503 |
| Abe | 6.8268 | 12.1569 |
| Tzambo | -0.9479 | 15.3679 |
| Porta | -1.4397 | 15.5599 |
| Oko | 7.3700 | 12.3500 |
| Katie (KB4986) | -1.4400 | 15.5600 |
| Choomba | 7.3700 | 12.3500 |
| Paki | 7.3700 | 12.3500 |
| Katie (B650) | -1.4400 | 15.5600 |

**Supplemental Figure 1:** Distribution of mtDNA haplogroups among the gorilla genomes used in the current study (after Soto-Calderon et al. 2015)
